# Ectodysplasin A/Ectodysplasin A Receptor System and Their Roles in Multiple Diseases

**DOI:** 10.3389/fphys.2021.788411

**Published:** 2021-12-06

**Authors:** Zhensheng Cai, Xia Deng, Jue Jia, Dong Wang, Guoyue Yuan

**Affiliations:** Department of Endocrinology, Affiliated Hospital of Jiangsu University, Zhenjiang, China

**Keywords:** ectodysplasin A, ectodysplasin A receptor, signaling pathways, metabolism, skeletal muscle homeostasis, tumorigenesis

## Abstract

Ectodysplasin A (EDA) is a member of the tumor necrosis factor (TNF) family of ligands that was initially reported to induce the formation of various ectodermal derivatives during normal prenatal development. EDA exerts its biological activity as two splice variants, namely, EDA-A1 and EDA-A2. The former binds to the EDA receptor (EDAR), resulting in the recruitment of the intracellular EDAR-associated death domain (EDARADD) adapter protein and the activation of the NF-κB signaling pathway, while the latter binds to a different receptor, EDA2R, also known as X-linked ectodermal dysplasia receptor (XEDAR). Inactivation mutation of the EDA gene or the genes coding for its receptors can result in hypohidrosis ectodermal dysplasia (HED), a condition that is characterized by oligotrichosis, edentulosis or oligodontia, and oligohidrosis or anhidrosis. Recently, as a new liver factor, EDA is gradually known and endowed with some new functions. EDA levels were observed to be upregulated in several metabolic diseases, such as non-alcoholic fatty liver disease (NAFLD), obesity, and insulin resistance. In addition, EDA and its receptors have been implicated in tumor pathogenesis through the regulation of tumor cell proliferation, apoptosis, differentiation, and migration. Here, we first review the role of EDA and its two-receptor system in various signaling pathways and then discuss the physiological and pathological roles of EDA and its receptors.

## Introduction

The ectodysplasin A (EDA) gene is a member of the tumor necrosis factor (TNF) family located on the long arm of the X chromosome. The EDA transcript encodes the EDA protein that generates several splice variants, two of which—EDA-A1 (391 amino acids) and EDA-A2 (389 amino acids) which contain a TNF homology domain (Kere et al., 1996; Park et al., 2019). EDA-A1 is a homotrimer type II transmembrane protein consisting of a transmembrane domain, a furan protease recognition site, and a 19-repeat Gly-X-Y collagen domain (Liu et al., 2019). EDA-A1 binds to the ectodysplasin A receptor (EDAR), which contains 14 cysteine residues, of which only the 6 closest to the N-terminus approximate the canonical TNF receptor consensus (Headon and Overbeek, 1999). EDA-A1/EDAR binding results in the recruitment of the intracellular EDAR-associated death domain (EDARADD) adaptor protein (Kumar et al., 2001) and the activation of the NF-κB signaling pathway (Sadia, Foo et al., 2019). Mutations in any of EDA, EDAR, and EDARADD can contribute to hypohidrotic ectodermal dysplasia (HED), which affects 1 in 10,000–100,000 newborns (Wohlfart et al., 2016; Feng et al., 2018; Liu et al., 2019). EDA-A2, which contains 2 amino acids less than EDA-A1, binds to a distinct receptor, EDA2R [also known as X-linked ectodermal dysplasia receptor (XEDAR)], indicating that the insertion of two amino acids into the ligand is a determinant of the specificity of receptor binding (Yan et al., 2000). Like EDA-A1, EDA-A2 also activates the NF-κB signaling pathway. TRAF6 can be recruited to the EDA-A2/EDA2R complex and thereby participate in the activation of the IκB kinase (IKK) complex, which is necessary for the translocation of NF-κB transcription factors into the nucleus (Yan et al., 2000). Studies have demonstrated that EDA-A2 is expressed in aging adipose, artery, heart, lung, muscle, and skin tissues and is associated with apoptosis (Yang et al., 2015; de Vries et al., 2017).

Although EDA and its receptors are known to be essential for ectodermal morphogenesis, their functions in disease pathology and related pathways are not well understood. EDA was recently identified as a liver-secreted protein involved in the occurrence and development of metabolic dysfunction. Loss- and gain-of-function studies have indicated that EDA, particularly the EDA-A2 isoform, regulates systemic glucose metabolism in type 2 diabetes mellitus (T2DM) (Awazawa et al., 2017). Additionally, the serum EDA-A2 level is dependent on T2DM, body mass index (BMI), and obesity (Yang et al., 2019). In patients with non-alcoholic fatty liver (NAFL) and non-alcoholic steatohepatitis (NASH), the plasma EDA content is high and is associated with deteriorating steatosis and fibrosis (Bayliss et al., 2021). In addition to the effects on HED and metabolic disorders, several studies have reported that EDA and its receptors are involved in cancer pathogenesis by regulating the apoptosis, proliferation, differentiation, and migration of cancer cells (Soraas and Stebbing, 2018; Vial et al., 2019; Li et al., 2020; Wang et al., 2020); however, this possibility remains controversial. Here, we review the role of EDA and its two-receptor system in various signaling pathways and then discuss the physiological and pathological roles associated with EDA and its receptors.

## Multiple Signaling Pathways Activated by Ectodysplasin A

Recent studies have demonstrated that EDA and its receptors participate in multiple signaling pathways, including the Wnt/β-catenin (Wang et al., 2020), c-Jun N-terminal kinase (JNK) (Sinha et al., 2002), bone morphogenetic protein (BMP)/Smad (Han et al., 2018), and fibroblast growth factor (FGF) signaling pathways (Häärä et al., 2012; Huh et al., 2013).

Wang et al. (2020) reported that EDAR promoted tumor cell proliferation by inducing Wnt/β-catenin signaling. The expression levels of genes related to the Wnt/β-catenin signaling pathway were upregulated in EDA^high^ samples. Additionally, the loss of EDAR could interfere with β-catenin signaling/luciferase activity, while silencing EDAR in colorectal cancer (CRC) cells led to a decrease in β-catenin abundance compared with that in vector shRNA-treated cells. Combined, these observations support that EDAR can activate the Wnt/β-catenin signaling pathway. Similarly, Zhang et al. (2009) found that Wnt/β-catenin signaling was necessary for the activation of the EDA/EDAR/NF-κB signaling pathway in epithelial cells, as well as the subsequent morphological and molecular events required for hair follicle development.

The EDA/EDAR/EDARADD pathway-mediated regulation of target genes is known to be dependent on the activation of the NF-kB pathway (Sadier et al., 2014); however, Sinha et al. (2002) reported that EDA2R can also activate the NF-κB and JNK pathways in an EDA-A2-dependent manner. Transient transfection of cDNA encoding FLAG-labeled XEDAR-L or XEDAR-s subtypes resulted in similar activation of the NF-κB pathway, while significant activation of NF-κB signaling was also observed in EDA-A2-treated 293F-XEDAR cells (Sinha et al., 2002). A different study demonstrated that EDA-A2 may activate NF-κB pathway by TRAF6. TRAF6 may be recruited to ligated XEDAR and contribute to activation of the IKK complex for translocation of NF-κB transcription factors into the nucleus (Yan et al., 2000). In addition to NF-κB activation, EDA-A2/EDA2R can also activate the JNK pathway by stimulating a rapid and marked increase in JNK1 and JNK2 phosphorylation, thereby promoting the phosphorylation-induced activation of the c-Jun transcription factor.

BMP2, BMP4, and BMP7 are expressed in early dental epithelial cells and are key regulators of tooth morphogenesis (Zouvelou et al., 2009; Feng et al., 2011; Jia et al., 2016). A recent study showed that in dental epithelial cells, EDA-A1 significantly induced Nkx2-3 expression in the pharyngeal floor as well as in oral cavity and branchial arch ectoderm (Biben et al., 2002). Han et al. (2018) found that BMP signaling is involved in tooth tip formation and tooth germ development by regulating cell differentiation and proliferation in the enamel node. Furthermore, Nkx2-3 transfection inhibited cell proliferation and induced the expression of Bmp2 and Bmpr2 mRNA and the phosphorylation of Smad1/5/8 in dental epithelial stem cells (M3H1 cells). These results indicated that Nkx2-3 was induced by EDA-A1 as a target molecule of the EDA-A1/EDAR pathway in dental epithelial cells and subsequently regulated cell proliferation through the BMP signaling pathway. The effects of BMP on EDAR have also been investigated. Mou et al. (2006) identified an EDAR/BMP activation-inhibition mechanism in which EDA-A1 upregulates EDAR expression, which subsequently induces BMPs expression, leading, in turn, to the suppression of EDAR expression. EDAR inhibited BMP rapidly and correlated with the level of phospho-Smad1/5/8, which is the activated form of intracellular transducers of BMP signals.

**FIGURE 1:**
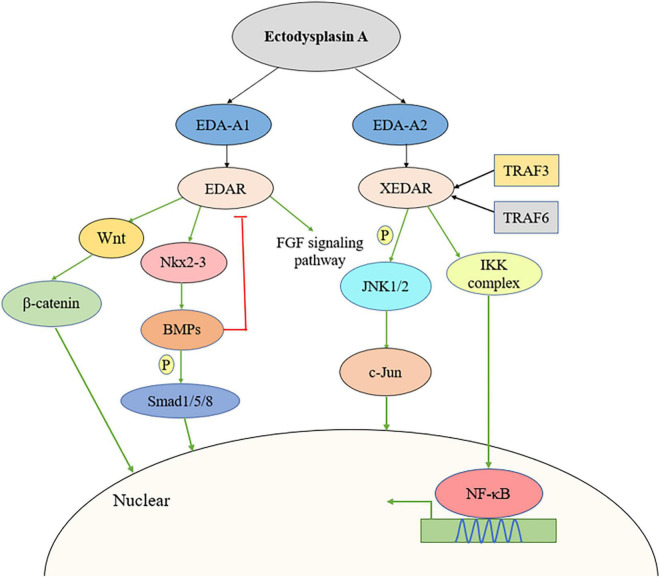
Multiple signaling pathways activated by EDA including Wnt/β-catenin signaling pathway, JNK pathway, BMP/Smad pathway, and FGF pathway. BMP, bone morphogenetic protein; FGF, fibroblast growth factor; JNK, c-Jun N-terminal kinase.

Microarray analysis showed that FGF20 is one of the earliest EDA-induced genes in hair placodes and, thus, is a putative transcriptional target of EDA (Fliniaux et al., 2008; Lefebvre et al., 2012). Häärä et al. (2012) demonstrated that FGF20 removal in EDA overexpression mice resulted in the appearance of a third molar, similar to the phenotype observed with EDA loss-of-function, suggesting that FGF20 has a critical role in modulating the initiation and size of posterior molars. The authors further found that EDA could rapidly induce FGF20 expression and FGF20 expression levels were related to EDA activity *in vivo*. Fgf20-null (Fgf20 ^βGal/βGal^) and Eda^–/–^ mice share similar molar phenotypes, indicating that FGF20 might be a key mediator and a direct target of EDA signaling transduction. Huh et al. (2013) revealed that, compared with untreated controls, the FGF20 message was increased by 3.3- and 16-fold after 2 and 4 h of EDA treatment, respectively. The activation of EDA in the epidermis led to an increase in FGF20^βGal^ activity, whereas the opposite was observed with the loss of EDA/EDAR signaling. Further analysis also indicated that the absence of FGF20 led to the suppression of EDAR expression and, consequently, also EDAR signaling (Figure 1).

## The Role of Ectodysplasin a in Hair Development

EDA is essential for the formation of skin appendages such as hair, teeth, sweat glands, and eyelids (Pinheiro and Freire-Maia, 1994). Recent studies have demonstrated that alopecia is independently associated with many concurrent conditions such as obesity, insulin resistance, and metabolic syndrome (Marks et al., 2019). HFD treatment can lead to hair loss in both young and aging mice, and multiple inductions of the hair cycle result in more severe and irreversible hair loss. Furthermore, HFD can accelerate both the anagen and telogen phases, shorten the hair growth cycle, and induce lipid droplet accumulation, ROS generation, and NF-κB signaling pathway activation in hair follicle stem cells (Morinaga et al., 2021). Liu et al. (2018) revealed that EDA mRNA and protein expression was higher in ear skin than in back skin. Moreover, alpaca ear hair is thick and straight with a long growth cycle, but its back hair is long, curved, and has a short growth cycle (Liu et al., 2011); these differences in growth cycle duration between the alpaca ear and back hair may be related to differential EDA expression. Kwack et al. (2019) explored the role of EDA-A2 and its receptor in hair follicles of mice and cultured human hair follicles. Compared with controls, the expression of the anti-apoptotic protein Bcl-2 was found to be decreased after rhEDA-A2 treatment, whereas that of the pro-apoptotic Bax and cleaved caspase-3 proteins was significantly increased. EDA2R mRNA expression peaked in the late anagen phase. Consistent with this, skin hair follicles injected with EDA-A2 showed catagen, compared with the anagen in skin injected with heat-inactivated EDA-A2. Similarly, Jiang et al. (2012) demonstrated that EDA mRNA expression in hair follicles of goat skin tissue was higher during the catagen stage than during the telogen and anagen phases. These results indicate that EDA can induce the apoptosis of hair follicle cells and regulate the hair follicle growth cycle. However, the relationship among obesity, EDA, and alopecia is not clear. Whether a HFD can lead to alopecia by affecting the expression of EDA need to be further explored, as do the associated regulatory mechanisms.

## The Role of Ectodysplasin a in Skeletal Muscle Homeostasis

Skeletal muscle is extremely important in regulating whole-body energy expenditure and determining resting energy expenditure. It is the main site for glucose metabolism, fatty acid oxidation, and insulin activity and an organ with high adaptability to environmental pressures, such as obesity (Zurlo et al., 1990; Stewart and Rittweger, 2006). The skeletal muscle is heterogeneous and consists of different fiber phenotypes of varying oxidative and glycolytic properties (Simcocks et al., 2020). Targeting skeletal muscle is a possible therapeutic strategy for improving metabolic homeostasis (Rossi et al., 2016). Newton et al. (2004) found that EDA-A2 might play a role in skeletal muscle homeostasis in an EDA2R expression-dependent manner. Myosin light-chain 2 (MLCH). EDA-A2 transgenic mice showed skeletal muscle degeneration in both weight-bearing and non-weight-bearing muscles. Importantly, EDA2R deficiency alleviated myodegeneration due to EDA-A2 overexpression. Additionally, a comparative analysis of EDA2R expression between skeletal muscle from wild-type and MLC2. EDA-A2 transgenic mice using an EDA2R riboprobe revealed that the EDA2R signal was strongest at sites of muscle damage in EDA-A2 transgenic mice. Recombinant human EDA-A2 can promote IκBα phosphorylation in normal human skeletal muscle cells, suggesting that EDA-A2 might cause muscle degeneration through IκBα phosphorylation; however, how EDA-A2 might exert these myodegenerative effects remains unknown. Studies have also demonstrated that treating 293E cells with recombinant human EDA-A2 can result in the activation of the IKK complex, leading to the phosphorylation of IκBα (Yan et al., 2000). In conclusion, EDA-A2 might instigate skeletal muscle degeneration by promoting IκBα phosphorylation through EDA2R. IκBα might be the downstream mechanism of EDA-A2, but whether it is involved in other diseases and deeper mechanisms need to be further explored.

## The Role of Ectodysplasin a in Metabolic Diseases

Glucose and lipid metabolism are closely linked and the stability of these two metabolic pathways is critical for maintaining body organ function (Du et al., 2021). Insulin resistance is highly associated with the occurrence and development of glucose metabolism disorder (James et al., 2021). Insulin resistance is defined as a reduction in the metabolic response of insulin-responsive cells to insulin or an impaired/reduced response of blood glucose levels to circulating insulin at the systemic level (Czech, 2017). In the liver, insulin not only regulates glucose production and utilization, but also has a broader influence on lipid metabolism (Chen, 2021). When circulating blood glucose levels are elevated, pancreatic β cells secrete insulin, which binds to hepatic insulin receptor (INSR). The receptor undergoes autophosphorylation, leading to the recruitment and phosphorylation of insulin receptor substrates (IRSs) that, in turn, activate downstream genes, finally resulting in AKT phosphorylation and activation. Once fully activated, AKT is involved in many downstream pathways, through which it regulates a variety of metabolic processes, including gluconeogenesis, glycolysis, glycogen synthesis, and lipid synthesis (Cherrington et al., 2007; Czech, 2017; Edgerton et al., 2017; Scherer, 2019). Insulin plays two main roles in the liver, namely, inhibiting glucose production (gluconeogenesis) and activating fatty acid and triglyceride (TG) synthesis (lipogenesis). Under the insulin resistance state, insulin does not inhibit gluconeogenesis; instead, it paradoxically over-activates adipogenesis, which leads to a fatal combination of hyperglycemia and hypertriglyceridemia (Staehr et al., 2004).

Insulin resistance in the liver is often accompanied by dyslipidemia and the occurrence of NAFLD (Byrne and Targher, 2015). The increase of fatty acid level is the main cause of hepatic steatosis and insulin resistance. Glucose feeding (and increased insulin levels) increases the production of new fat and stimulates the expression, nuclear localization, and transcriptional activity of sterol regulatory element-binding transcription factor 1 (SREBP1c) and other transcription factors (Horton et al., 2002). After activation of SREBP1c, the expression of genes related to *de novo* lipid synthesis, such as fatty acid synthase (FAS) and acetyl-CoA carboxylase (ACC), is upregulated, thereby increasing the production of fatty acids (Horton et al., 2002; Alves-Bezerra and Cohen, 2017). Peroxisome proliferator-activated receptor gamma (PPARγ), another nuclear hormone receptor, contributes to energy storage primarily by promoting adipogenesis and lipid synthesis and displays the highest expression levels in white adipose tissue (WAT); meanwhile, the complete transcriptional activity of PPARs requires the binding of homologous lipid ligands and heterodimerization with retinoid-X receptor (RXR), also a nuclear receptor. PPARγ phosphorylation may restore insulin sensitivity by enhancing PPARγ function. In contrast, PPARγ dominant-negative mutations result in hypertension and insulin resistance, suggesting that a relationship exists between PPARγ function and metabolic syndrome (Plutzky, 2011; Christofides et al., 2021).

Several studies have shown that EDA, a recently identified hepatokine, is mainly expressed in the liver and can be secreted into the circulatory system to participate in energy and glycolipid metabolism (Awazawa et al., 2017; Yang et al., 2019; Bayliss et al., 2021). Awazawa et al. (2017) found that the expression levels of EDA, corresponding to miR-676, were higher in the livers of *db/db* mice than in those of control mice. Clinical studies have shown that in humans, EDA expression in the liver is positively correlated with liver fat content, visceral fat area, and NASH scores. Additionally, EDA expression was significantly decreased after surgery, and was accompanied by weight loss and improved insulin sensitivity. A case-control study (Yang et al., 2019) showed that the serum EDA-A2 concentration in patients with NAFLD was higher than that in the controls. The frequency of NAFLD increased with increasing EDA-A2 levels. ROC curve analysis also revealed that EDA-A2 levels could predict the presence of NAFLD. Significant and positive associations were found between EDA-A2 levels and BMI, waist-to-hip ratio (WHR), fasting plasma glucose (FPG), hemoglobin A1c (HbA1c), and homeostasis model assessment of insulin resistance (HOMA-IR), a series of anthropometric parameters and some parameters of glucose metabolism and insulin function. In addition, Bayliss et al. (2021) found that liver EDA mRNA levels were higher in NASH group than in those without NAFLD; however, no difference was detected between NAFL and non-NAFLD patients. Compared with patients without NAFLD, plasma EDA concentrations were increased in both the NAFL and NASH groups and were positively correlated with the degree of steatosis. Interestingly, the authors proposed that plasma EDA was not a reliable biomarker for NAFLD and could not discriminate between NAFL and NASH. Several reasons can explain the differences between these two studies. First, Yang et al. assessed serum EDA-A2, while Bayliss et al. assessed total serum EDA. EDA-A1 and EDA-A2 share a TNF homologous domain and differ by only two amino acids. Moreover, the proportion of EDA-A2 in total EDA is unclear. The second main reason relates to the different criteria used for NAFLD diagnosis. Yang et al. used ultrasonography and graded NAFLD based on the Chinese Standard, while in Bayliss’s study, NAFLD was determined using liver biopsy and histological assessment. In conclusion, it remains unknown whether EDA concentrations can predict the presence of NAFLD, and additional studies are required to address the above-described discrepancies.

In experiments *in vivo*, evaluation based on enzyme-linked immunosorbent assay (ELISA) showed that the plasma EDA concentrations were higher in *db/db* mice and obese mice than in their respective control groups (Awazawa et al., 2017). EDA-A2 overexpression resulted in higher glucose concentration in the glucose tolerance test and lower energy consumption relative to control (GFP-AAV-injected) mice. Immunoblot analyses showed a comparable upregulation of insulin receptor substrate 1 (IRS1) phosphorylation at Ser307 in skeletal muscle. Similarly, suppressing EDA expression decreased blood glucose concentrations in an insulin tolerance test but did not influence weight, energy expenditure, exercise ability, or food intake. Furthermore, a different study (Yang et al., 2019) showed that EDA knockdown attenuated hepatic lipogenesis in HepG2 cells. The TG content in free fatty acid (FFA) + EDA small interfering RNA (siRNA)-treated cells was significantly lower when compared with that in cells treated with FFAs alone. The effect of EDA on liver lipid metabolism might be exerted through the regulation of lipolysis- and lipogenesis-related genes such as SREBP1c and the key fatty acid synthesis-associated enzymes FAS and ACC. The TG content of mice fed a high-fat diet (HFD) for 8 weeks was significantly increased, while the HFD-induced increase in the numbers of lipid droplets was markedly weakened in EDA-depleted mice. In addition, EDA knockdown inhibited serum aspartate transaminase (AST) and alanine transaminase (ALT) activity, but not that of alkaline phosphatase (ALP).

The mechanism involved in how obesity leads to the upregulation of EDA expression in the liver has also been investigated. Awazawa et al. (2017) found that the combined expression of PPARγ and RXR-α induced EDA promoter activity and EDA mRNA expression in Hepa1-6 cells, while the overexpression of either factor alone or the pharmacological activation of PPARγ failed to induce EDA expression in the liver. JNK is one of the best-characterized signal transducers in obesity and insulin resistance (Pal et al., 2016; Solinas and Becattini, 2017). *In vivo* administration of EDA-A2 increased JNK phosphorylation, while JNK phosphorylation levels were found to be higher in mice injected with EDA-A2-AAV than in control mice (Awazawa et al., 2017).

**FIGURE 2:**
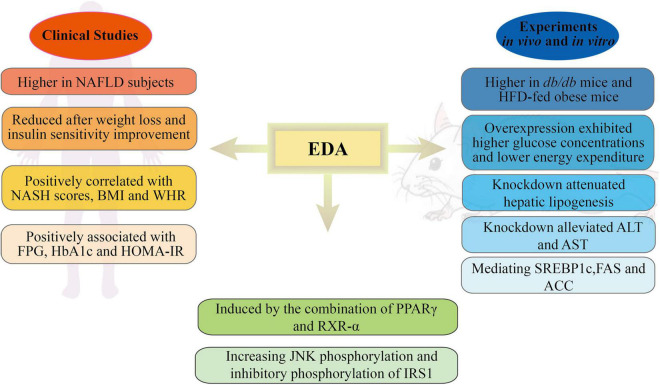
Schematic representation of the roles and mechanisms of EDA in metabolic disorders. NAFLD, non-alcoholic fatty liver disease; NASH, non-alcoholic steatohepatitis; BMI, body mass index; WHR, waist-to-hip ratio; FPG, fasting plasma glucose; HbA1c, hemoglobin A 1c; HOMA-IR, homeostasis model assessment of insulin resistance; HFG, high-fat diet; ALT, alanine transaminase; AST, aspartate transaminase; SREBP1c, sterol regulatory element binding transcription factor 1; FAS, fatty acid synthase; ACC, acetyl-CoA carboxylase; PPARγ, proliferator-activated receptor γ; RXR-α, retinoid-X receptor-α; IRS1, insulin receptor substrate 1.

Collectively, these findings suggest that EDA secreted from liver tissues is associated with insulin resistance, diabetes, and NAFLD and that stimulating EDA or blocking EDA signaling may modulate hepatic steatosis and insulin resistance; however, the specific underlying mechanisms remain elusive (Figure 2). In addition, although some studies have shown that EDA-A2 has a role in metabolic diseases, other studies have been unable to distinguish whether this regulation is exerted by EDA-A1 or EDA-A2. This highlights the need to investigate the impact of EDA-A1 and EDA-A2 on metabolic disorders separately, including the detection of their concentrations in circulating blood and their expression levels in liver tissues, as well as their specific effects using loss- and gain-of-function methods.

## The Role of Ectodysplasin a in Diabetic Nephropathy

Diabetic nephropathy (DN) is a common complication in patients with diabetes. It is the main cause of chronic kidney disease (CKD) and end-stage renal disease (ESRD). DN is characterized by podocyte apoptosis, mesangial cell proliferation, matrix expansion, and glomerular and tubulointerstitial fibrosis (Wang et al., 2021). Studies have indicated that EDA may have a role in the development of DN. The expression of EDA2R has been reported to be upregulated in diabetic kidneys (in both type 1 and type 2 diabetes) (Watanabe et al., 2013; Brennan et al., 2018). Recently, one study also showed that the mRNA and protein expression of EDA2R was increased in both type 1 (STZ injection) and type 2 (*Btbr* ^ob/ob^) diabetic mice relative to controls. Consistent with the results in animals, EDA2R was also found to be highly expressed in podocytes treated with high glucose concentrations *in vitro* as well as in glomerular podocytes of diabetic patients. Moreover, EDA2R might provoke podocyte injury through the generation of reactive oxygen species (ROS). EDA2R overexpression was shown to inhibit the expression of anti-apoptotic molecules such as Mcl-1 and Bcl-2 while enhancing that of pro-apoptotic molecules such as Bax and cleaved caspase-3. EDA2R increased ROS production in podocytes, while inhibiting ROS generation could weaken EDA2R-mediated podocyte injury. In addition, EDA2R-knockdown podocytes displayed attenuated ROS production under stimulation with high glucose concentrations. The silencing of EDA2R expression partially relieved the occurrence of high glucose-induced apoptosis and dedifferentiation (Lan et al., 2020). EDA2R might exacerbate the development of DN by regulating the apoptosis and dedifferentiation of podocytes and enhancing the generation of ROS. It is not clear whether similar mechanisms mediate the involvement of EDA2R in glycolipid metabolism and DN or whether EDA2R can serve as a novel therapeutic target for the treatment of patients with DN.

## The Role of Ectodysplasin a in Tumorigenesis

Although EDA has been proposed to play a regulatory role in cell proliferation and differentiation, this possibility remains controversial and warrants further investigation. For instance, the epidermal growth factor receptor (EGFR) gene is one of the most frequently mutated genes in lung cancer, particularly in non-small cell lung adenocarcinoma (Rosell et al., 2009). Soraas and Stebbing (2018) identified a significant and positive correlation between EDAR polymorphism and EGFR mutation frequencies, indicating that the EDAR gene might be associated with lung cancer and may have potential as a biomarker for the diagnosis of this disease. In a different study (Wang et al., 2020), the authors showed that the mRNA and protein expression of EDAR was upregulated in CRC tissues and CRC cell lines relative to their respective controls. Additionally, CRC patients with high EDAR expression have poor clinical outcomes, whereas those with low EDAR expression showed improved overall survival rates. Compared with vector controls, shRNA-mediated knockdown of EDAR significantly reduced the size and number of CRC cell colonies and induced cell cycle arrest in the G1 phase. *In vivo*, the tumor burden of mice transplanted with shEDAR-transduced tumor cells was significantly alleviated, and the tumor volume of EDAR-deficient mice was less than 1,000 mm^3^. In addition, Li et al. (2020) showed that EDARADD was highly expressed in head and neck squamous cell carcinoma (HNSCC) tissues while EDARADD expression was associated with the degree of tumor differentiation and local recurrence in tongue squamous cell carcinoma (TSCC). Furthermore, EDARADD knockdown in TSCC cells affected clonogenicity, induced apoptosis, suppressed proliferation, and reduced the expression of NF-κBp65, MYC, and Bcl-2. NF-κB plays a broad role in cell proliferation and mediates apoptosis, especially its RelA (p65) subunit (Giridharan and Srinivasan, 2018; Zeng et al., 2019). MYC is located downstream of NF-κB and promotes cell growth and proliferation, while Bcl-2 family proteins regulate apoptosis (Adams and Cory, 1998; Zhao et al., 2018). These results all demonstrate that both EDAR and EDARADD are involved in cancer pathogenesis; in contrast, however, Vial et al. (2019) revealed that EDAR acts as a tumor suppressor in melanoma. The authors observed a marked decrease in EDAR expression in malignant melanoma compared with that in benign nevi. Each EDAR mutation (T167I, E254K, P409L, and V416M) significantly impaired EDAR pro-apoptotic activity. EDAR knockout mice were reported to develop melanoma lesions over the first 400 days, a phenotype that was linked with reduced survival. Together, these findings suggest that EDA is closely linked with cancer cell proliferation, migration, differentiation, and local recurrence. Whether EDA exerts positive or negative effects is likely to be dependent on tumor type, and requires further investigation.

## Conclusion

In summary, EDA, a hepatokine, may be associated with a variety of diseases, such as metabolic disorders, DN, and cancer, and may be a link among insulin resistance, T2DM, obesity, and NAFLD. Our review highlighted the potential roles of this hepatokine and the possibility of targeting the EDA signaling pathway in different pathophysiological processes. Importantly, elevated circulating EDA levels are closely associated with the incidence of NAFLD, rendering EDA a potential biomarker for the clinical diagnosis of this condition. However, further clinical investigation is warranted to confirm this possibility.

## Data Availability Statement

The original contributions presented in the study are included in the article/supplementary material, further inquiries can be directed to the corresponding authors.

## Author Contributions

ZC and GY conceived and designed the review. ZC and XD analyzed the data and wrote the original draft of the manuscript. DW and JJ revised the final manuscript. All authors read and approved the final manuscript.

## Conflict of Interest

The authors declare that the research was conducted in the absence of any commercial or financial relationships that could be construed as a potential conflict of interest.

## Publisher’s Note

All claims expressed in this article are solely those of the authors and do not necessarily represent those of their affiliated organizations, or those of the publisher, the editors and the reviewers. Any product that may be evaluated in this article, or claim that may be made by its manufacturer, is not guaranteed or endorsed by the publisher.
